# Amyloid mis-metabolism in idiopathic normal pressure hydrocephalus

**DOI:** 10.1186/s12987-016-0037-y

**Published:** 2016-07-29

**Authors:** A. Jeppsson, Mikko Höltta, H. Zetterberg, K. Blennow, C. Wikkelsø, Mats Tullberg

**Affiliations:** 1 Hydrocephalus Research Unit, Institute of Neuroscience and Physiology, The Sahlgrenska Academy, University of Gothenburg, 413 45 Gothenburg, Sweden; 2 Department of Psychiatry and Neurochemistry, Institute of Neuroscience and Physiology, the Sahlgrenska Academy, University of Gothenburg, Gothenburg, Sweden; 3 Clinical Neurochemistry Laboratory, Sahlgrenska University Hospital, Molndal, Sweden; 4 Department of Molecular Neuroscience, UCL Institute of Neurology, Queen Square, London, UK

**Keywords:** Neurodegeneration, Normal pressure hydrocephalus, Cerebrospinal fluid, Amyloid, NFL, YKL40, Prediction, APLP1

## Abstract

**Background:**

Patients with idiopathic normal pressure hydrocephalus (iNPH) have reduced cerebrospinal fluid (CSF) concentrations of amyloid-β (Aβ) and α- and β-cleaved soluble forms of amyloid precursor protein (sAPPα and sAPPβ). The aims of this study were to examine if changes could also be seen in the CSF for secreted metabolites of APP-like protein 1 (APLP1) and to explore the prognostic value of amyloid-related CSF biomarkers, as well as markers of neuronal injury and astroglial activation, as regards to clinical outcome after shunt surgery.

**Methods:**

Twenty patients diagnosed with iNPH, 10 improved and 10 unchanged by shunt surgery, and 20 neurologically healthy controls were included. All patients were examined clinically prior to surgery and at 6-month follow-up after surgery using the iNPH scale. Lumbar puncture was performed pre-operatively. CSF samples were analyzed for neurofilament light (NFL), Aβ isoforms Aβ38, Aβ40 and Aβ42, sAPPα, sAPPβ, APLP1 β-derived peptides APL1β25, APL1β 27 and APL1β 28 and YKL40 by immunochemical methods.

**Results:**

The concentrations of all soluble forms of APP, all Aβ isoforms and APL1β28 were lower, whilst APL1β25 and APL1β27 were higher in the CSF of iNPH patients compared to controls. There was no difference in biomarker concentrations between patients who improved after surgery and those who remained unchanged.

**Conclusions:**

The reduced CSF concentrations of Aβ38, Aβ40, Aβ42, sAPPα and sAPPβ suggest that APP expression could be downregulated in iNPH. In contrast, APLP1 concentration in the CSF seems relatively unchanged. The increase of APL1β25 and APL1β27 in combination with a slight decreased APL1β28 could be caused by more available γ-secretase due to reduced availability of its primary substrate, APP. The data did not support the use of these markers as indicators of shunt responsiveness.

## Background

Idiopathic normal pressure hydrocephalus (iNPH) is a condition with gait and balance disturbances, cognitive decline and urinary incontinence in combination with enlarged cerebral ventricles [1, 2]. Shunt treatment improves more than 80 % of the patients [3]. Without surgery, the clinical course is progressive and a delay in treatment means a loss of function that cannot be restored [4]. Being one of the few treatable neurodegenerative conditions, an accurate diagnosis and identification of patients who will benefit from shunt surgery is essential. The use of CSF biomarkers for such purposes has been identified as one of the priorities for hydrocephalus research [5].

Patients with iNPH exhibit suppressed CSF concentrations of amyloid-β (Aβ) and the precursors soluble amyloid precursor protein α-, and β- (sAPPα, sAPPβ), in combination with elevated neurofilament light protein (NFL) [6–9]. Hypothetically, this is thought to be due to a downregulation of APP in the periventricular tissue possibly caused by changed amyloid metabolism and/or a reduced clearance of extracellular fluid into CSF leading to lowered concentrations of APP-derived proteins in CSF [6].

Amyloid-like protein 1 (APLP1)-derived peptides are processed by similar enzymatic pathways as APP and share related structural domains and functions [10–12]. APLP1 is processed into short Aβ-like peptides (APL1β25, 1β27 and 1β28) [13]. APLP1 is a substrate for the enzyme γ-secretase and the ratio of APLP1-derived APL1β28 to total APL1β is a surrogate marker for Aβ42 production in the central nervous system [13, 14]. Recently, it was reported that γ-secretase was higher in brain biopsies from iNPH patients with amyloid plaques than in those without [15].

The aim of this study was to examine CSF concentrations of APLP1-derived peptides in iNPH, especially if the APL1β28 form was increased, and to explore the prognostic value of amyloid-related CSF biomarkers. For this purpose, we analyzed the APP-derived peptides sAPPα, sAPPβ, Aβ38, Aβ40 and Aβ42, the APLP1derived peptides APL1β25, APL1β27 and APL1β28 in CSF in 20 patients with iNPH (10 improved and 10 unchanged by shunt operation) and 20 neurologically healthy controls.

## Methods

### Study populations

Ten iNPH patients improved after shunt surgery and 10 non-improved were retrospectively selected. All were diagnosed in accordance with the international guidelines [16]. The patients were selected from our local database at the hydrocephalus unit at Sahlgrenska University Hospital on the premises that full medical data pre- and postoperatively were available and that there was sufficient CSF stored to perform the analyses. In all, the database contained 176 patients. From the database, the 10 patients who benefitted the most from surgery (as defined by improvement in the iNPH scale) and fulfilled the inclusion criteria were selected. In the group that did not benefit from surgery, medical records were scanned in order to establish that at time for follow-up all shunts were working, none had complications and that that there be no other known cause for non-improvement. Patients who had complications that could be attributed to shunt surgery were excluded. In all, the groups were selected in order to magnify the difference in responsiveness to shunt surgery within the clinical material and analyze two clearly-distinguishable extreme groups as regards to shunt responsiveness.

The improved 10 patients consisted of five men and five women, aged 70.3 ± 3.20 (mean ± SD) and the 10 unimproved patients (<5 points at the iNPH scale) consisted of seven men and three women aged 71.6 ± 8 (mean ± SD). The groups did not differ significantly in terms of comorbidities, preoperative MMSE scores, extent of white matter lesions (WML), age or sickness duration. The baseline clinical data of the different groups are outlined in Table 1.

**Table 1:** Clinical characteristics of iNPH patients at baseline

	Improved	Non-improved	
	n = 10	n = 10	
Age (mean ± SD)	70.3 ± 3.2	71.6 ± 8.0	ns
Sex (male/female)	5/5	7/3	ns
Sickness duration (month)	42 ± 21	34 ± 28	ns
Diabetes (y/n)	2/8	2/8	ns
Hypertension (y/n)	5/5	6/4	ns
Cardiovascular disease (y/n)	2/8	1/9	ns
MMSE (median, IQ-range)	23 (22–28)	26 (24–28)	ns
WML (median, IQ-range)	6 (4–10)	11 (5–20)	ns
Evans index (median, IQ-range)	0.43 (0.38–0.46)	0.39 (0.36–0.41)	ns

All patients were examined clinically prior to surgery and 6 months after by the iNPH scale, composed of items assessing gait, cognition, continence and balance [17]. The extent of WML was rated by the Wahlund scale from MRI or CT scans at the time for diagnosis [18]. Lumbar puncture was performed preoperatively with the patient in recumbent position. In the improved group the median improvement was 26 points and in the non-improved group the median was 1 point (Table 2). All patients received a ventriculo-peritoneal shunt with a programmable valve with an anti-siphon device and a Rickham reservoir. All had working shunts and none had complications at the time for evaluation.

**Table 2:** iNPH scale score pre op, post op and outcome (median and IQ-range)

	Pre op		Post op		Outcome	
	Improved	Non-improved	Improved	Non improved	Improved	Non-improved
	n = 10	n = 10	n = 10	n = 10	n = 10	n = 10
Gait domain	33 (16–48)	54 (35–69)	84 (57–100)	50 (34–81)	49 (26 to 57)	−1 (−6 to 11)
Cognitive domain	64 (37–73)	60 (46–80)	75 (57–85)	69 (48–80)	10 (6 to 21)	3 (−6 to 11)
Continence domain	60 (20–80)	80 (60–80)	90 (75–100)	70 (55–80)	30 (0 to 45)	0 (−20 to 5)
Balance domain	67 (67–71)	67 (67–83)	75 (67–87)	67 (67–83)	0 (−4 to 20)	0 (−16 to 0)
Total iNPH score	50 (36–64)	63 (56–70)	77.3 (71–87)	64 (52–71)	26 (21 to 30)	1 (−3 to 3)

Twenty control subjects were chosen from a population of volunteers who had given consent to CSF sampling prior to knee surgery. The controls had no history of neurological or psychiatric disease, a normal clinical neurological examination and a normal mini-mental state examination score. They consisted of eight men and 12 women aged 71.2 (±6.4). There was no difference in age between the three subcategories (controls, iNPH improved and iNPH non-improved).

### CSF analyses

Amyloid β isoforms (Aβ38, Aβ40, and Aβ42) were analyzed by electrochemiluminescence assays (Meso Scale Discovery, Gaithersburg, MD, USA). The APLP1-derived peptides APL1β25, APL1β27, and APL1β28 were analyzed using a commercial ELISA (IBL International, Hamburg, Germany). The samples were analyzed according to the kit insert with minor modifications. The CSF samples were diluted 1:20 for APL1β25, 1:10 for APL1β27, and 1:5 for APL1β28 by the dilution buffer contained in the kit. All samples were analyzed in duplicate and the CV % for standards and samples was <5 %.

NFL was measured by ELISA technology using a commercial kit (NF-Light, UmanDiagnostics, Umeå, Sweden) with a lower limit of detection of 50 ng/L. For astroglia activation, CSF YKL-40 concentration was measured by solid phase sandwich ELISA (R&D Systems, Inc., Minneapolis, Minnesota, USA) according to the manufacturer’s instructions. All analyses were performed batch-wise on one occasion by board-certified laboratory technicians at the Clinical Neurochemistry Laboratory at Sahlgrenska University Hospital, Mölndal, Sweden. Intra-assay coefficients of variation were below 10 %.

### Statistics

Non-parametric methods were used for analyses. For comparisons between two groups the Mann–Whitney U test was performed and for comparisons between the three subgroups, the Kruskal–Wallis test was performed. For comparison of two proportions, Fisher’s exact test was used. For association between two independent variables, the Spearman rank order correlation was chosen. The level of significance chosen was *p* = 0.05, if not otherwise stated. No correction for the mass-significance effect was made in order to avoid type II errors. Statistical analyses were made using IBM^®^ SPSS^®^ Statistics for Windows version 21.

## Results

**Fig. 1:**
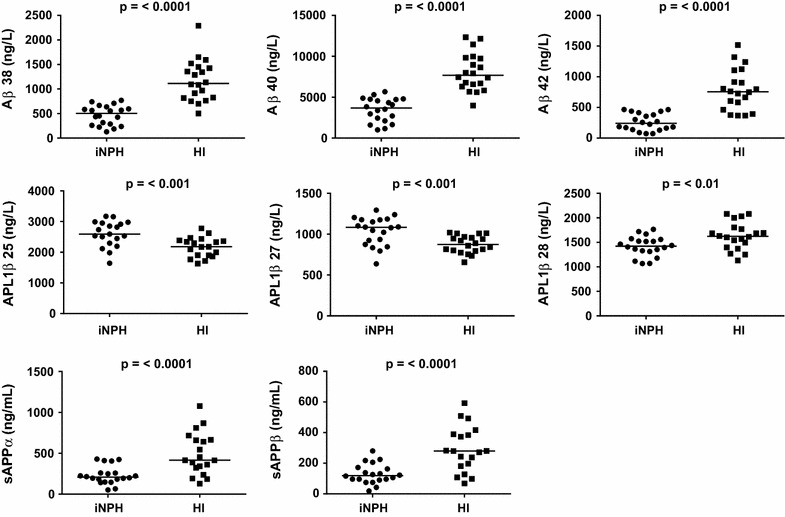
Scatter plot showing CSF concentrations of Amyloids (Aβ38, 40, 42), APLP1 derivates (APL1β25, 27, 28) and amyloid precursor proteins (sAPPα, β) in 20 patients with iNPH and 20 controls. Medians are indicated by horizontal lines. P values indicate the level of significance. The differences between the difference in concentrations of APLP1-derived proteins are, although statistically significant, more modest

**Fig. 2:**
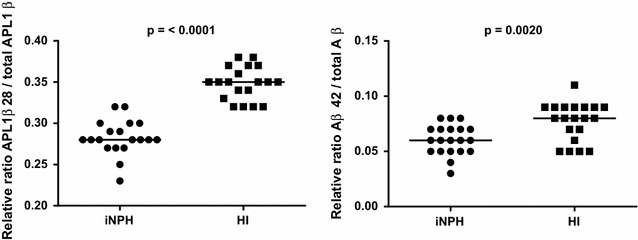
The relative ratios of APL1β28 to total APL1β and Aβ42 to total Aβ in CSF in 20 patients with iNPH and 20 Controls. P values show the level of significance

CSF concentrations of sAPPα, sAPPβ, Aβ38, Aβ40, Aβ42 and APL1β28 were significantly lower and APL1β25 and 27 significantly higher in iNPH patients compared to healthy controls. Levels of NFL and YKL 40 did not differ between iNPH patients and healthy controls (Table 3; Fig. 1). The APL1β28/total APL1β ratio and the Aβ42/to total Aβ ratio was lower in patients with iNPH in comparison with healthy controls (Fig. 2).

**Table 3:** Biomarker levels in iNPH and controls (median and IQ range)

	iNPH	Controls
	n = 20	n = 20
NFL (ng/L)	1185 (731–2103)	938 (610–2141)
APL1β25 (ng/L)	2591 (2296–2951) ↑	2180 (1898–2386)***
APL1β27 (ng/L)	1083 (887–1177) ↑	874 (796–964)***
APL1β28 (ng/L)	1423 (1317–1550) ↓	1621 (1422–1797)**
Aβ38 (ng/L)	502 (266–625) ↓	1114 (819–1445)***
Aβ40 (ng/L)	3676 (2190–4748) ↓	7682 (6366–9809)***
Aβ42 (ng/L)	241 (144–405) ↓	754 (493–1058)***
sAPPα (ng/mL)	207 (157–259) ↓	416 (323–665)***
sAPPβ (ng/mL)	119 (92–170) ↓	280 (182–389)***
YKL40 (ng/mL)	122 (90–167)	137 (104–177)

Arrows indicating biomarker levels in iNPH in comparison with controls

*ns* non-significant

* *p* ≤ 0.05; ** *p* ≤ 0.01; *** *p* ≤ 0.001

There were no differences in any of the CSF biomarkers between improved and non-improved iNPH patients (Table 4).

**Table 4:** Levels of biomarkers in improved vs non-improved iNPH patents (median and IQ-range)

	Improved	Non-improved	
	n = 10	n = 10	
NFL (ng/L)	1186 (869–1670)	1085 (699–2432)	ns
APL1β25 (ng/L)	2532 (2174–2958)	2820 (2401–2954)	ns
APL1β27 (ng/L)	1067 (900–1157)	1085 (867–1214)	ns
APL1β28 (ng/L)	1423 (1264–1568)	1458 (1291–1562)	ns
Aβ38 (ng/L)	500 (308–605)	503 (224–677)	ns
Aβ40 (ng/L)	3731 (2642–4740)	3677 (1522–4789)	ns
Aβ42 (ng/L)	241 (155–370)	244 (122–438)	ns
sAPPα (ng/mL)	205 (175–279)	212 (144–297)	ns
sAPPβ (ng/mL)	114 (95–155)	127 (75–181)	ns
YKL40 (ng/mL)	122 (99–153)	134 (84–180)	ns

*ns* non-significant

## Discussion

Our data showing substantially reduced CSF concentrations of Aβ38, Aβ40, Aβ42, sAPPα and sAPPβ in patients with iNPH, confirm earlier results [6–9, 11, 19–22]. In contrast, APLP1 in CSF was mildly but significantly, changed with an increase in APL1β25 and APL1β27 and a slight decrease of APL1β28, compared with controls. The ratios APL1β28/total APL1β and Aβ42/total Aβ were reduced in patients with iNPH. However, there were no differences in any CSF biomarker between improved and non-improved iNPH patients after treatment with shunt surgery.

This explorative study was in part designed to identify predictive alterations in the amyloid CSF pattern. We included two small groups representing iNPH patients that benefitted the most and those without any response to surgical treatment with shunting, in order to identify possible differences. However, we found no differences in CSF biomarkers between improved and non-improved patients. The low number of cases in each group may have been a limitation in this study. However, if there are subtle variances in CSF amyloid concentrations linked to responsiveness that could be identified in larger sample sets, we believe that these would be of minor clinical importance. Lumbar CSF as opposed to ventricular CSF was analysed to enhance the practical applicability of the generated results. Ventricular CSF might however provide a different pattern as it probably mirrors brain metabolic processes more accurately.

The reduced CSF concentrations of Aβ38, Aβ40, Aβ42, sAPPα and sAPPβ in iNPH is consistent with earlier findings [6, 7, 9, 11, 19–23], and compatible with a reduction in the concentrations of APP-derived proteins in the CSF of iNPH-patients. iNPH is a disorder of disturbed CSF dynamics and/or consequences thereof. Recent insights into the glymphatic system, has provided possibilities for a new route for clearance of excess fluid and interstitial metabolites, including Aβ, from the brain parenchyma [24, 25], together with clearance of Aβ across the blood–brain barrier (BBB) via the LRP-1 receptor [26]. In the glymphatic system, there may be a para arterial influx of CSF from the subarachnoid space into the brain parenchyma where convective flow of interstitial fluid (ISF) helps to clear metabolic waste by para venous clearance towards the cervical lymph system, a system that seems to impair with aging [25, 27]. However, clear evidence for this clearance is still lacking. In iNPH the CSF flow above the convexities is reduced and redirected into the ventricles and iNPH patients often present with a disproportionately enlarged subarachnoid-space [28, 29]. These findings could hypothetically be in accordance with a disturbance of CSF/ISF exchange as the dilated paravascular spaces could impair CSF/ISF exchange. Although speculative, it could be hypothesized that the reduced concentrations of APP-derived proteins in the CSF of INPH patients could be due to stagnation of the flow in the periventricular ISF with reduced clearance of Aβ. This, however, remains to be proven. Regardless, levels of APP-derived proteins in CSF are affected by both production and clearance of APP, and also by the ISF/CSF itself. This could make estimates of tissue levels in relation to CSF concentration problematic in patients with iNPH [30].

CSF APP-derived proteins increase after surgery, more in improved patients than in those that do not improve [6, 11]. Even if not designed to analyze such changes, the present study does not contradict these findings. The presumed reduction in CSF concentrations of the amyloid-derived proteins could reflect a pathophysiological aspect of iNPH that is not directly linked with prediction. The irreversibility in non-improved iNPH patients could be due to either tissue damage related to iNPH or damage related to other factors such as co-morbidities e.g. cerebrovascular lesions, as there was a tendency of more profound WML in the non-improved group even if not reaching statistical significance. However, cerebrovascular disease is not a negative predictor of outcome after shunt surgery [31].

Contrary to the profound alterations in APP metabolites, the APLP1-derived peptides showed only minor changes with a small elevation in APL1β25 and APL1β27 and a slight reduction of APL1β28. APP and APLP1 are processed by the same enzymes, including γ-secretase [10, 12, 13, 32], and the results could indicate that the two substrates compete with each other at the active site of γ-secretase. If APP expression is reduced, as most data suggest it is in iNPH, there would be more γ-secretase available for the processing of APLP1. The processing occurs by γ-secretase cleaving at amino acid 28 of the membrane-spanning β-domain of APLP1 and then working its way towards the N-terminus of the protein. Increased processing of APLP1 by γ-secretase would thus result in decreased concentration of APL1β and increased concentrations of the shorter forms [33, 34]. This is exactly what we observe in iNPH. Over-expression of APP results in a decrease of APL1β, which supports this substrate competition hypothesis [35].

There is a difference in APP-metabolite production pattern between iNPH and AD. In iNPH there is a general suppression of APP-metabolites in CSF whereas in AD, there is an isolated Aβ42 reduction, whereas the other Aβ-isoforms are unaffected [36]. Moreover, the internal composition of Aβ production differs as shown in the APL1β28/total APL1β ratio. In our opinion, this provides further evidence against a common pathological etiology and might aid in the differential diagnosis of iNPH from AD by CSF biomarkers [37].

## Conclusions

This data lends further support to a diagnostic profile in iNPH consisting of a general reduction in CSF concentration of APP-derived proteins. That the amyloid-like proteins behave in a different pattern could support the specificity and importance of the APP-down-regulation in iNPH. The study indicates that the biomarker profile in iNPH is consistent between patients who improve by shunt insertion and those who do not; therefore, our results do not lend support to the idea that these markers can be used for predictive purposes, but rather as an aid in the diagnosis of iNPH. Further studies will be needed to replicate the results and to expand the knowledge on the role of a possible altered amyloid metabolism for the pathogenesis of iNPH and the potential use of markers of amyloid metabolism to identify shunt responders needs to be further elucidated.

## Abbreviations

iNPH: idiopathic normal pressure hydrocephalus
CSF: cerebrospinal fluid
Aβ: amyloid-β
sAPPα: α-cleaved soluble forms of amyloid precursor protein
sAPPβ: β-cleaved soluble forms of amyloid precursor protein
APLP1: APP-like protein 1
APL1β: APLP1 β-derived Aβ-like peptide
NFL: neurofilament light
WML: white matter lesion
ISF: interstitial fluid
BBB: blood–brain barrier
